# Effectiveness of an integrated approach to reduce perinatal mortality: recent experiences from Matlab, Bangladesh

**DOI:** 10.1186/1471-2458-11-914

**Published:** 2011-12-10

**Authors:** Anisur Rahman, Allisyn Moran, Jesmin Pervin, Aminur Rahman, Monjur Rahman, Sharifa Yeasmin, Hosneara Begum, Harunor Rashid, Mohammad Yunus, Daniel Hruschka, Shams E Arifeen, Peter K Streatfield, Lynn Sibley, Abbas Bhuiya, Marge Koblinsky

**Affiliations:** 1 Centre for Reproductive Health, icddr,b, Mohakhali, Dhaka 1212, Bangladesh; 2 School of Human Evolution and Social Change, Arizona State University, P.O. Box 872402, Tempe, AZ 85287-2402, USA; 3 Emory University, 1520 Clifton Road, NE, Atlanta, GA 30322-4027, USA; 4 John Snow Inc, 1776 Massachusetts Avenue, Suite 300, Washington, DC, USA

## Abstract

**Background:**

Improving perinatal health is the key to achieving the Millennium Development Goal for child survival. Recently, several reviews suggest that scaling up available effective perinatal interventions in an integrated approach can substantially reduce the stillbirth and neonatal death rates worldwide. We evaluated the effect of packaged interventions given in pregnancy, delivery and post-partum periods through integration of community- and facility-based services on perinatal mortality.

**Methods:**

This study took advantage of an ongoing health and demographic surveillance system (HDSS) and a new Maternal, Neonatal and Child Health (MNCH) Project initiated in 2007 in Matlab, Bangladesh in half (intervention area) of the HDSS area. In the other half, women received usual care through the government health system (comparison area). The MNCH Project strengthened ongoing maternal and child health services as well as added new services. The intervention followed a continuum of care model for pregnancy, intrapartum, and post-natal periods by improving established links between community- and facility-based services. With a separate pre-post samples design, we compared the perinatal mortality rates between two periods--before (2005-2006) and after (2008-2009) implementation of MNCH interventions. We also evaluated the difference-of-differences in perinatal mortality between intervention and comparison areas.

**Results:**

Antenatal coverage, facility delivery and cesarean section rates were significantly higher in the post- intervention period in comparison with the period before intervention. In the intervention area, the odds of perinatal mortality decreased by 36% between the pre-intervention and post-intervention periods (odds ratio: 0.64; 95% confidence intervals: 0.52-0.78). The reduction in the intervention area was also significant relative to the reduction in the comparison area (OR 0.73, 95% CI: 0.56-0.95; *P *= 0.018).

**Conclusion:**

The continuum of care approach provided through the integration of service delivery modes decreased the perinatal mortality rate within a short period of time. Further testing of this model is warranted within the government health system in Bangladesh and other low-income countries.

## Background

Reduction of perinatal mortality remains a major public health concern in low- and middle-income countries including Bangladesh [1-3]. Of approximately 8.8 million under-five child deaths, 40% are estimated to take place during the neonatal period (first 4 weeks of life) globally [4]. In addition to neonatal deaths, about 2.6 million stillbirths (fetal deaths after 28 weeks of gestation) occur each year, which have similar consequences as neonatal death for family members, but remain largely unseen in official statistics [5-9]. Improvement of neonatal health, especially during the early neonatal period when about 60-80% of the deaths occur, is crucial to achieving the Millennium Development Goal (MDG) 4 for child survival [10]. Reviews suggest that scale up of the known evidence-based interventions following a life-cycle continuum (pregnancy, delivery and post-natal periods) and provided through integrated service delivery modes (family/community, outreach, and facility-based) may impact significantly on fetal and neonatal survival [11,12].

Community-based trials with varying packages of maternal and neonatal interventions have been successful in reducing stillbirth and neonatal mortality in South Asia. Home-based interventions with outreach by trained community health workers in rural areas, including standardized assessment and treatment of neonatal infections, has reduced perinatal mortality in India [13] and neonatal deaths in Bangladesh [14]. However, a similar community-based package coupled with facilitation of referral to health facilities in Mirzapur, Bangladesh did not have any effect on perinatal survival [15]. Community-based studies which educated women through women's groups about effective newborn interventions using pictorial cards have reduced perinatal mortality in both Nepal [16] and India [17]. However, a study using similar interventions in northern Bangladesh did not observe any effect on perinatal mortality [18]. While community-based interventions are promising, integrating these strategies with accessible quality services at facility levels is likely to have a much greater effect in improving perinatal health. As reported by Darmstadt et al. integrated community- and facility-based service delivery modes can potentially decrease neonatal mortality by about 40-70% [19,20]. The objective of this study was to evaluate the effect of an integrated maternal-newborn health program using community- and facility-based approaches on perinatal mortality in a rural area in Matlab, Bangladesh.

## Methods

### Study site

The study was conducted in Matlab, a sub-district of Chandpur district, where the International Centre for Diarrhoeal Disease Research, Bangladesh (icddr, b) has implemented a health and demographic surveillance system (HDSS) in a population of about 220,000. Since 1966, community health research workers (CHRWs) have collected vital statistics such as marriage, birth, death, and migration. The HDSS area is divided into two parts: the icddr, b service area (SA) and the government SA. The icddr, b SA is divided into 4 administrative blocks, each serving a population of about 27,000 through a sub-center staffed by midwives that provide 24-h delivery care. Clinical activities in the sub-centers are supported by an icddr, b hospital in Matlab Township that offers basic obstetric care, staffed by medical doctors and nurses.

In the icddr, b SA, a maternal-child health and family planning (MCH-FP) program was initiated in 1977. The program started with distribution of family planning methods to women of reproductive age and provision of services for minor illnesses to women and children. Over the years, services were added to improve maternal and child health. In 1987, a safe motherhood program was introduced to increase the coverage of home births by midwives posted at the sub-center levels. A transport system was available for referral of women from sub-centers to the Matlab Hospital. The home-birth strategy continued until 1996, after which it was redesigned for facility-based delivery care. Between 1996 and 2001, all four sub-centers of icddr, b SA were upgraded and equipped to perform basic obstetric care; home births with midwives were no longer offered.

In the government SA, women receive pregnancy, delivery, and post-natal care from government health facilities. However, other non-formal health care providers (such as traditional birth attendants, village doctors) are active in the area. The lowest level of health care facility is the Health and Family Welfare Centre (union level sub-centre), which is equivalent to icddr, b sub-center, and provide antenatal and delivery care through a paramedic and a medical assistant. These sub-centers are linked with Upazila Hospital located at Matlab municipality where medical doctors, nurses and other support staff are available and provide essential obstetric care.

As both of the hospitals (icddr, b and Upazila) only provide basic obstetric care, woman who needs cesarean section has to travel to the neighboring districts--Chandpur and Narayanganj located in the south and the north of Matlab, respectively. The government district hospital and a growing number of private clinics provide such care. Women from icddr, b SA and from the south part of the government SA usually travel to Chandpur, which is about 20 km away from Matlab proper. The women from the northern part of the government service area usually travel to Narayanganj, which is 3-4 h journey by boat or by bus.

### Study design and population

Initiated in 2007, the Maternal, Neonatal and Child Health (MNCH) Project was embedded in the ongoing MCH-FP Project. The MNCH Project implemented evidence-based maternal and neonatal interventions following the continuum of care approach from pregnancy to delivery to the postnatal period, and improving links between community- and facility-based service delivery modes.

The study employed a separate pre-post samples design which compared change in perinatal outcomes in the icddr, b SA and the government SA. Changes in study outcomes were assessed between two time periods--before (2005-2006) and after (2008-2009) implementation of MNCH intervention program in the icddr, b SA. We also used a difference-of- differences analysis to evaluate the reduction in perinatal mortality in the icddr, b SA (served as an intervention area) relative to the reduction in the government SA (served as a comparison area). We excluded outcome data from 2007, the year the interventions were rolled out.

The study population included pregnant women identified through routine bimonthly CHRWs' surveillance visits to households, and study participants were women who gave birth between 2005 and 2009 (excluding 2007). In total, 20,766 women were included in the analyses (n = 10,659 from icddr, b SA and n = 10,107 from government SA).

To evaluate the knowledge and practice on newborn care, we also conducted a baseline and an end-line survey in November 2006-February 2007 and April to June 2010, respectively, on randomly selected women delivered in the last 1 year in both icddr, b SA and government SA. In total 4,704 women were interviewed from both areas.

### Interventions and process of implementation of study

**Figure 1:**
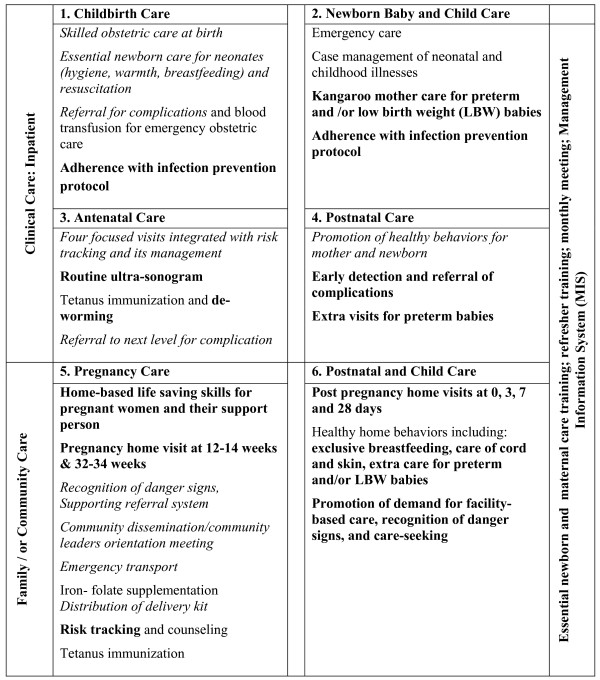
**Interventions in place following the continuum care life (pregnancy/ANC, childbirth and post-partum) and place of service delivery (community/outreach, outpatient and facility based) with the Maternal, Neonatal, and Child Health (MNCH) project in Matlab 2007-2009**. Font-weight: normal - interventions unchanged; italic: interventions strengthened; bold: new interventions added (adopted from the model by Kerber at al. 2007) [12]

The MNCH Project aimed to increase facility-based delivery and to improve maternal, neonatal and child health. At the outset of the project, a needs assessment focusing on training, infrastructure, and administrative issues was conducted. Based on this assessment, priority areas were selected--including strengthening existing interventions under the existing MCH-FP program and adding new interventions as needed. Figure 1 displays the full intervention package differentiating between the existing MCH-FP interventions (before the present study) and new interventions using the model presented by Kerber et al. [12]. Interventions were described in two dimensions: the life cycle and mode of service delivery (family/household, outreach, facility). We also included a third dimension on quality assurance and management information system at all levels (Figure 1).

#### Community level interventions

In 2007, CHRWs were separated into surveillance and service CHRWs. The surveillance CHRWs were responsible for pregnancy identification and collection of vital event information including pregnancy outcomes. The service CHRWs were responsible for all curative and preventive care at community levels, including household visits.

Pregnant women were identified during routine bi-monthly surveillance CHRWs' visits and confirmed by spot urine test. At 12-14 weeks of pregnancy, service CHRWs visited the pregnant woman at home, and if the woman consented to participate in the study, provided counseling on danger signs, referral and birth planning using a pictorial card. Service CHRWs also encouraged each woman to select a support person to assist her with labor and delivery, and counseled the woman and her family on the importance of attending four facility-based antenatal care visits.

At weeks 32-34, a service CHRW visited the woman and her family for a second time to provide specific messages on birth planning, newborn care and to establish a birth team. During this visit, CHRWs encouraged pregnant women to attend a facility for delivery. After delivery, CHRWs conducted home-based postnatal visits within 24 h of delivery (for women who gave birth at home), and on days 3, 7, and 28 (for all women). During these postnatal visits, CHRWs identified danger signs using an algorithm based on the guidelines outlined in the WHO manual for child, neonatal and maternal health [21], and referred mother or neonate to the Matlab hospital if needed, provided counseling on newborn and maternal wellbeing, breastfeeding, and family planning.

Home-based Life Saving Skills (HBLSS), developed by the American College of Nurse-Midwives, was an important component of the intervention package [22]. HBLSS is a family- and community-focused, competency-based training for women and their identified support persons on prevention, first aid and referral for post-partum hemorrhage, prolonged labor and birth asphyxia [23]. The content is reinforced through a pictorial Take Action Card that is taken home for reference [24]. Each pregnant woman and her support person were encouraged to participate in 4 HBLSS sessions during pregnancy. These group-sessions were conducted by the service CHRWs at the respective CHRW's home.

In addition to the interventions, there were bi-weekly meetings at each sub-centre attended by CHRWs, midwives from the respective sub-centre and MNCH project staff to discuss problems identified at field and facility levels and to provide refresher trainings on the ongoing interventions.

#### Facility level interventions

Standard guidelines were developed and implemented for the management of maternal and newborn complications, including mouth-to-mouth and bag-and-mask ventilation to resuscitate babies with asphyxia. At both the icddr, b sub-center and hospital levels, providers' skills were strengthened on management of normal and complicated deliveries as well as management of newborn complications through a series of trainings conducted within icddr, b and outside involving tertiary care hospitals (Institute of Child and Maternal Health, Matuail, Dhaka; and Bangladesh Institute of Research and Rehabilitation in Diabetes, Endocrine and Metabolic Disorders Hospital, Dhaka, Bangladesh).

The MNCH project offered four focused ANC visits either at a sub-centre or at icddr, b Matlab Hospital covering physical examination, risk identification and management, and counseling including birth preparedness. All deliveries in icddr, b facilities were monitored by the partograph and active management of the third stage of labor using oxytocin was routinely practiced. Support persons were allowed in the delivery room, and women could select their labor position. Women requiring cesarean section were referred to the Chandpur district hospital or a private clinic where the MNCH Project had established lower fees for intervention area (icddr, b service area) patients. Referral transport was provided by the project. In addition the project constructed a Kangaroo Mother Care (KMC) unit adjacent to Matlab Hospital for care of low birth weight and/or premature babies.

At icddr, b Matlab hospital, services were further strengthened to provide magnesium sulfate for pre-eclampsia and eclampsia, assisted deliveries (with vacuum), manual removal of the placenta, blood transfusion, standardized management of post-date pregnancies through protocol, corticosteroid treatment for women with preterm labor, and antibiotic use with premature rupture of membranes.

In addition, quality assurance was in place with a checklist for ANC, infection prevention, delivery care, immediate newborn care and Kangaroo Mother Care activities at the facility level. The checklist was conducted bi-monthly by a medical officer, who was also part of the research team. Any discrepancy in service delivery observed was discussed with the health care provider and continually observed until corrected accordingly. The community level HBLSS sessions and CHRW pregnancy and postnatal home visits were also checked by the assigned paramedical staff for each intervention block. We also established perinatal audits in both hospital and community levels. To improve the quality of care, the factors related with administration, logistics and quality of care generated from the perinatal audits were discussed with all staff during the monthly meeting of the MNCH program. In addition a web-based hospital management system was in place that followed selected process and outcome indicators related with the project; this information guided the projects staff to strengthen the project efforts where gaps were noted.

### Care in the government service area

The government of Bangladesh (GoB) took initiative to improve the development of human resources and infrastructure (through comprehensive emergency obstetric care, basic emergency obstetric care, and birthing centers) and to upgrade the skills of skilled birth attendants. However, the available services provided through facilities are not specified well. The survey reported that the important elements of antenatal care such as providing iron supplements, educating women on the signs of pregnancy complications, performing screening tests (including urine and blood tests), and measuring weight gain and blood pressure were not uniform at population level across different strata of the society [25]. Overall, the coverage of these interventions is also low. There are lack of routine ultrasound examination, risk tracking, management protocol for preterm delivery, post-term pregnancies, and thermal care of neonates immediate after delivery in the government health facilities. Considering the low coverage of ANC and skilled delivery practices, the GoB has started a new initiative--Demand Side Financing (DSF) in several Upazilas (sub-districts) all over the country. The government SA has been a part of this initiative since 2008. With this program women receive some incentives for ANC care and delivery by skilled personnel. In addition service providers also get remuneration for their efforts [26].

### Outcome and related data

Outcome information was collected during bi-monthly surveillance CHRWs' visits, including pregnancy outcomes (spontaneous abortion, induced abortion, stillbirth, live birth) and survival during the neonatal period. Spontaneous abortion was defined as unintended loss of the fetus in the first 28 weeks of gestation, as determined by the last reported menstrual period. Induced abortion was defined as intended loss of fetus in the first 28 weeks of gestation. Stillbirth was defined as birth of a dead fetus after 28 weeks of gestation. Live birth was defined as birth of a fetus with any sign of viability. Neonatal death was defined as the death of a live-born baby before 28 days of age. Early neonatal death was defined as death of neonates within 7 days of birth of a live-born baby. Perinatal death was defined as fetal (stillbirth) or early neonatal death. For each woman, delivery information such as place of delivery and type of delivery was also collected.

Information on cause of death was collected using the verbal autopsy method. Using a modified structured questionnaire developed by the World Health Organization [27], interviews were conducted with the caretakers/relatives who had lived with the infant in the same household during the terminal stages of illness and death of the infant. A physician reviewed each verbal autopsy form and filled out death certificates using the International Classification of Diseases version 10 (ICD10) codes, with notes to support the diagnosis.

Detailed information on women's age, parity, education, and household assets were collected from the HDSS databases and confirmed during interviews with study participants. Parity was defined as number of live or dead children before the current pregnancy. Educational status was assessed as number of years completed at school. Economic status was assessed by generating scores through principal-components analysis based on household assets, housing structure, land occupation, and income. These scores were then indexed into quintiles, where 1 represented the poorest and 5 the richest [28]. Last menstrual period date was determined by recall during the pregnancy-identification interview at the monthly household visits. Gestational age at pregnancy outcome was measured by subtracting the last menstrual period date from date of pregnancy outcome and was expressed in weeks.

### Statistical analysis

Perinatal mortality, before and after the intervention, was analyzed using logistic regression in both icddr, b and government SA. The period before intervention (2005-2006) was used as the reference and associations were presented using odds ratios with their 95% confidence intervals. We also determined the difference-of-differences in perinatal mortality before and after the intervention between the icddr, b SA and government SA. We coded the time period as before = 0 and after = 1, and the area as government SA (comparison) = 0 and icddr, b SA (intervention) = 1, and made an interaction term (time × area). The time, area and interaction term were entered in the logistic regression model. The alpha-value for the interaction term was set at 0.05 to indicate whether the reductions between two areas were statistically different. We also, divided the study area according to geographic importance into north and south, and determined odds of perinatal mortality between two time periods.

Background characteristics were evaluated with outcome variables and association determined by chi-square, analysis of variances, the Wald test, or by non-parametric test depending on the type of data being analyzed. Any socio-demographic characteristics associated with outcome variables at *P *< 0.20 significance level were included in the logistic regression model for adjustment.

### Ethical considerations

The study strengthened ongoing maternal and child health services and added evidence-based interventions at both community and facility levels. All women from icddr, b SA gave consent for participation in the MNCH study. In addition we also used routine data collected by the ongoing HDSS. This study was approved by the Research and Ethical Review Committees of International Centre for Diarrhoeal Disease Research, Bangladesh.

## Results

Of the 10,659 births included in the study from the icddr, b SA, 10,412 resulted in live births (97.7%), 247 resulted in stillbirths (23/1000 births) and 178 resulted in early neonatal deaths (17/1000 live births). In the government SA, of the 10,107 births, 9,793 resulted in live births (96.9%), 314 resulted in stillbirths (31/1000 births), and 264 resulted in early neonatal deaths (27/1000 live births).

The background characteristics of the study participants before and after the intervention in both icddr, b and government SAs are presented in Table 1. The socio-demographic characteristics, although significantly different between the two periods, were more or less similar in two areas. The proportion of preterm births (birth before 37-week gestation) in post-intervention period was significantly lower compared with pre-intervention period in both areas (Table 1).

**Table 1:** Background Characteristics of Study Women Before (2005-2006) and After (2008-2009) Intervention Implementation by Service Area, Matlab, Bangladesh

Variables*	icddr, b Service Area			Government Service Area		
	
	Before	After	*P*-value	Before	After	*P*-value
				
	N (%)	N (%)		N (%)	N (%)	
Age(years)						
< 20	749 (14.0)	778 (14.7)	0.015	703 (13.3)	563 (11.7)	0.019
			
20-24	1742 (32.5)	1755 (33.1)		1691 (32.0)	1633 (33.9)	
			
25-34	2285 (42.7)	2297 (43.3)		2358 (44.6)	2095 (43.5)	
			
> = 35	578 (10.8)	475 (9.0)		539 (10.2)	525 (10.8)	
			
Mean (SD†) age in year	26.5 (6)	26.2 (6)		26.5 (6)	26.7 (6)	

Parity						

0	1997 (37.3)	2058 (38.7)	< 0.001	1840 (34.8)	1761 (36.6)	0.001
			
1-2	2524 (41.1)	2612 (49.3)		2389 (45.2)	2226 (46.2)	
			
> = 3	833 (15.6)	634 (12.0)		1062 (20.1)	829 (17.2)	

Asset index						

1	843 (15.7)	821 (15.5)	0.244	888 (16.8)	749 (15.6)	< 0.001
			
2	927 (17.3)	904 (17.0)		1037 (19.6)	805 (16.7)	
			
3	1108 (20.7)	1070 (20.2)		1033 (19.5)	1100 (23.0)	
			
4	1227 (22.9)	1171 (22.1)		1214 (22.9)	1092 (22.7)	
			
5	1249 (23.3)	1339 (25.2)		1119 (21.1)	1060 (22.0)	

Education (years)						

No education	1075 (20.1)	1007 (19.0)	0.188	1036 (19.6)	1313 (27.3)	< 0.001
			
Primary	1384 (25.8)	1440 (27.1)		1559 (29.5)	1396 (29.0)	
			
Secondary or above	2895 (54.1)	2858 (53.9)		2696 (51.0)	2107 (43.8)	

Gestational age (weeks)						

< 37	897 (16.8)	650 (12.3)	< 0.001	905 (17.1)	714 (14.8)	< 0.001
			
> = 37	4457 (83.2)	4655 (87.3)		4386 (82.9)	4102 (85.2)	
			
Mean (SD†) gestational age in weeks	38.8 (2.6)	39.1 (2.4)		38.7 (2.5)	39.0 (2.5)	

*All are presented in percent unless stated

†*SD*: standard deviation

The majority of women in the icddr, b SA were exposed to the MNCH interventions (Table 2). About 94% of women received home visits during early pregnancy (gestational week 12-14), 77% during late pregnancy (gestational week 32-34). In the icddr, b SA, ANC visits were almost universal (78% of women received 3 or more ANC) during the intervention period in comparison with the pre -intervention period (38%). Although statistically significant, the increase in ANC care was not pronounced in the government SA between two periods of observations. Marked increase in facility deliveries (from 55% to 72% in icddr, b SA, and from 13% to 22% in the government SA) and cesarean section rates (from 8% to 16% in icddr, b SA, and from 5% to 10% in the government SA) were observed between the two periods in both areas (Table 2).

**Table 2:** Coverage of Selected Indicators Before (2005-2006) and After (2008-2009) Intervention Implementation by Service Area, Matlab, Bangladesh

Variable Name	icddr, b Service Area			Government Service Area		
	
	Before	After	*P*-value	Before	After	*P*-value
				
	Count (%)	Count (%)		Count (%)	Count (%)	
1st Pregnancy Home visit (12-14 weeks)						

No	N/A	305 (5.7)	N/A*	N/A	N/A	N/A
			
Yes	N/A	5000 (94.3)		N/A	N/A	

2nd Pregnancy Home visit (32-34 weeks)						

No	N/A	1236 (23.3)	NA	N/A	N/A	N/A
			
Yes	N/A	4069 (76.7)		N/A	N/A	

PNC Home visits						

No	N/A	964 (18.2)	N/A	N/A	N/A	N/A
			
Yes	N/A	4341(81.8)		N/A	N/A	

HBLSS sessions						

0	N/A	415 (7.8)	N/A	N/A	N/A	N/A
			
1	N/A	271 (5.1)		N/A	N/A	
			
2	N/A	451 (8.5)		N/A	N/A	
			
> = 3	N/A	4115 (78.7)		N/A	N/A	

Facility ANC visit						

0	245 (4.5)	225 (4.2)	< 0.001	834 (15.7)	760 (15.6)	0.005
			
1	810 (15.1)	327 (6.2)		2134 (40.3)	1782 (37.0)	
			
2	2276 (42.5)	616 (11.6)		1984 (37.5)	1912 (39.7)	
			
> = 3	2023 (37.8)	4137 (78.0)		362 (7.5)	362 (7.5)	

Place of delivery						

Home	2425 (45.3)	1465 (27.6)	< 0.001	4583 (86.6)	3717 (78.2)	< 0.001
			
Facility delivery	2929 (54.7)	3840 (72.4)		708 (13.4)	1034 (21.8)	

Delivery type						

Vaginal	4889 (91.2)	4466 (84.2)	< 0.001	5009 (94.7)	4329 (89.9)	< 0.001
			
Cesarean	418 (7.8)	839 (15.8)		282 (5.3)	487 (10.1)	

*N/A*: not application; *PNC*: postnatal care; *HBLSS*: home-based live saving skills; *ANC*: antenatal care

High risk practices such as early bathing and late initiation of breast feeding were significantly different between the base-line and end-line periods in both icddr, b and government SA. Knowledge on newborn care practices also increased, however the changes were more pronounced in the icddr, b SA (Table 3).

**Table 3:** Immediate Newborn Care Practices and Knowledge of Newborn Danger Signs Before (2005-2006) and After (2008-2009) Intervention Implementation by Service Area, Matlab, Bangladesh

	icddr, b service area			Government service area		
	
	Baseline (n = 1211) (%)	End-line (n = 1153) (%)	*P*-value	Baseline (n = 1196) (%)	End-line (n = 1144) (%)	*P*-value
Newborn care practice						

Timing of first newborn bath						

< 1 day	29.7	4.1	< 0.001	43.7	23.6	< 0.001
			
1 day	8.8	2.0		6.7	6.8	
			
2 days	14.2	8.1		7.0	11.2	
			
> = 3 days	45.2	84.6		41.3	56.1	
			
Don't remember	2.1	1.2		1.3	2.3	

Breastfeeding practice						

Colostrums as first food						

Yes	82.5	96.1	< 0.001	57.8	75.3	< 0.001
			
No	17.5	3.9		42.2	24.7	

Timing of breastfeeding after birth						

< 30 min	60.9	80.9	< 0.001	45.8	58.1	< 0.001
			
30-59 min	21.0	6.7		23.4	20.3	
			
> = 60 min	18.2	12.4		30.8	21.7	

Knowledge of danger sign for newborn						

Difficult or fast breathing	90.4	91.2	0.26	82.4	81.5	0.28

Poor sucking or feeding	20.8	31.7	< 0.001	12.5	9.8	0.02

Yellow coloration skin/eye	41.0	29.0	< 0.001	34.4	28.8	0.002

Pus discharge from umbilical cord	7.9	44.3	< 0.001	3.8	4.4	0.26

Pus discharge from eyes	2.7	25.2	< 0.001	0.8	1.0	0.37

Very small baby	4.6	15.2	< 0.001	1.8	1.8	0.55

Convulsion	33.9	49.0	< 0.001	17.1	20.8	0.01

Lethargy/unconsciousness	1.7	2.8	0.05	0.7	0.8	0.46

None of above/others	2.6	1.4	0.02	6.4	7.5	0.17

Women's age, parity, education, asset index, and gestational age were associated with perinatal mortality in both areas (Table 4). Antenatal care coverage was significantly associated with perinatal mortality in the icddr, b SA but not in the government SA (Table 4).

**Table 4:** Association of Background Characteristics with Perinatal Mortality by Service Area in Matlab, Bangladesh

Variables	icddr, b Service Area	Government Service Area
	
	Odds ratio (95% confidence interval)	Odds ratio (95% confidence interval)
Age in years		

< 20	1.12 (0.81-1.54)	0.94 (0.71-1.24)

20-24*	1.00	1.00

25-34	1.14 (0.90-1.44)	0.84 (0.69-1.02)

> = 35	2.05 (1.52-2.78)	1.52 (1.17-1.97)

Parity		

0*	1	1

1-2	052 (0.42-0.64)	0.36(0.29-0.43)

> = 3	0.66 (0.49-0.90)	0.53 (0.42-0.67)

Asset index		

1 Poorest	1.34 (0.98-1.84)	1.33 (1.01-1.74)

2	1.15 (0.83-1.58)	0.96 (0.72-1.27)

3	1.30 (0.96-1.74)	1.23 (0.95-1.59)

4	1.18 (0.88-1.59)	1.07 (0.82-1.38)

5 Richest*	1.00	1.00

Education in year		

0	1.59 (1.26-2.01)	1.40 (1.14-1.71)

1-5	1.06 (0.83-1.35)	1.04 (0.85-1.28)

> 5*	1.00	1.00

Gestation age in weeks		

< 37	4.87 (3.98-5.95)	3.37 (2.82-4.03)

> = 37*	1.00	1.00

No. of antenatal care visits		

= < 1	3.03 (2.37-3.90)	1.19 (0.83-1.71)

2	2.33 (1.86-2.92)	1.15 (0.79-1.65)

> = 3*	1.00	1.00

Delivery Place		

Home	0.90 (0.73-1.10)	0.43 (0.36-0.52)

Facility*	1.00	1.00

Type of delivery		

Cesarean section	1.05 (0.77-1.43)	1.13 (0.84-1.53)

Vaginal *	1.00	1.00

*reference category

**Figure 2:**
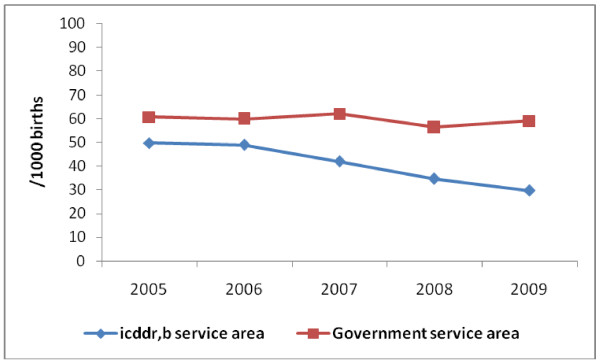
**Perinatal Mortality Rate by Year in the Study Area 2005-2009 in Matlab, Bangladesh**.

Perinatal mortality decreased from 48/1000 births to 32/1000 births, respectively before and after the interventions in the icddr, b SA (Figure 2). The initial reduction of perinatal mortality in 2007 reflected the improvement of knowledge and skills of health care providers and initiation of evidence based practices at facility levels in a short period of time. There was almost no change in mortality in the government SA. The adjusted odds of perinatal mortality after the intervention was 36% lower (OR: 0.64; 95% CI: 0.52-0.78) compared with the rate before the intervention period (Table 5) in the intervention area (icddr, b SA). In the same area, when we stratified the analyses by place of delivery we found the odds of perinatal mortality (adjusted for women's age, parity, education and asset index) at icddr, b facility, home and other facilities were 0.69 (95% CI: 0.50-0.95), 0.75 (95% CI: 0.53-1.08) and 0.35 (95% CI: 0.23-0.52), respectively in the post-intervention period in comparison to the odds of perinatal deaths among women delivered during the pre-intervention period. The perinatal mortality rates were similar in both periods in the government SA (Table 5).

**Table 5:** Association of Perinatal Mortality with Intervention Periods, Before (2005-2006) and After (2008-2009) Intervention Implementation, by Service Area in Matlab, Bangladesh

	**Birth No**.	Perinatal death No. (%)	Odds ratio (95% CI)	Adjusted odds ratio* (95% CI)
icddr, b Service Area				

Before†	5354	257 (4.8)	1.00	1.00

After	5305	168 (3.2)	0.65 (0.53-0.79)	0.64 (0.52-0.78)

Government Service Area				

Before†	5291	309 (5.8)	1.00	1.00

After	4816	269 (5.6)	0.95 (0.81-1.13)	0.88 (0.74-1.04)

CI: confidence interval

†reference group

*Adjusted for women's age, parity, education, and asset index in quintiles

We observed significant interaction between area of intervention and time-period (*P *= 0.018). There was 27% relative reduction in the odds of perinatal mortality in icddr, b SA compared to the reduction observed in the government SA (OR 0.73, 95% CI: 0.56-0.95).

When we divided the population in different groups based on geographic location, we also observed similar trends in odds of perinatal mortality. In the icddr, b SA, the odds of perinatal mortality were 0.54 (95% CI: 0.39, 0.74) and 0.69 (95% CI:0.54, 0.89), respectively, in north and south after the intervention in comparison to the period before. The similar figures in north and south part of the government SA were 0.91 (95% CI: 0.70, 1.18) and 0.90 (95% CI: 0.72, 1.13), respectively.

Birth asphyxia, prematurity, and infections were the leading causes of death as determined by verbal autopsies in both areas. We observed marked decrease in deaths related with birth asphyxia and infections in the icddr, b area (Table 6).

**Table 6:** Cause Specific Early Neonatal Mortality Rate (death/1000 live births) During the Study Periods, Before (2005-2006) and After (2008-2009) Intervention Implementation, by Service Area in Matlab, Bangladesh

Causes of deaths	icddr, b Service Area		Govt. Service Area	
	
	Before n (rate*)	After n(rate*)	Before (Rate) n (rate*)	After (Rate) n (rate*)
Asphyxia	44 (8.4)	26 (5.0)	68 (13.3)	50 (10.7)

Infections	13 (2.5)	6 (1.5)	12 (2.3)	17 (3.6)

Preterm/growth retarded	20 (3.8)	21 (4.0)	31 (6.0)	31 (6.6)

Malformation	5 (1.0)	9 (1.7)	1 (0.2)	1 (0.2)

Other causes	26 (5.0)	8 (1.5)	32(6.2)	21 (5.0)

Total	108	70	144	120

*per 1000 live births

## Discussion

Perinatal mortality remains an important public health issue in Bangladesh and other low-income settings. We show that an integrated intervention package across the continuum of care and provided at all levels of the service delivery system--home, out-reach clinic and facility level was associated with a 36% reduction in perinatal mortality. This finding has public health significance not only as a first, in terms of provision of an integrated package at all levels of service delivery, but also considering the observed effect in an area with a relatively low rate of perinatal mortality, and achieved in a short timeframe (a 2-3 year frame). Services were delivered at home/outreach clinics by CHRWs, at sub-centers by midwives and at hospital level mainly by midwives with support from medical officers with the possibility of referral but without the presence of specialists in obstetrics or neonatology.

The present study has several strengths. In addition to its large sample size, it involved continuous data collection over a 2 year period which diminishes the effects of seasonal variation on perinatal mortality. A separate group of CHRWs recorded both stillbirth and early neonatal death to prevent misclassification of outcomes. Relevant socio-demographic information serves as a statistical control on confounding. Moreover, the use of pre-post measurements and a comparison area as well as the existence of perinatal mortality data for numerous years prior to intervention provide important checks on internal validity.

However, the study results should be interpreted cautiously. As the intervention included integrated service delivery at multiple levels and also implemented evidence-based interventions, a randomized trial was not feasible. The baseline differences in ANC coverage, facility delivery, and cesarean section rates between the two areas limit the comparability between areas after implementation of the intervention. This difficulty in comparability between Matlab study areas at baseline in determining the final outcome has also been noted in other studies [29]. This raises several potential questions to the study finding's validity, some of which can be addressed by examining longer term trends in perinatal mortality. First, the observed effect of interventions on perinatal mortality may have resulted from a natural decrease over the time period of the study. However, we did not observe any change in perinatal mortality in the 2 years prior to the initiation of the MNCH project in either the icddr, b SA or government SA. Once the project began, perinatal mortality declined sharply (Figure 2). When we divided the study site into several areas with physical importance (north and south), we also observed similar pattern of perinatal mortality before and after the intervention periods. Furthermore, in a previous study in the same area it was reported that the decline in stillbirth and neonatal deaths was about 24% and 39%, respectively over a period of 28 years (1975-2002) [30]. In the present study, a similar reduction in perinatal mortality was achieved in a 2-year period. The temporal specificity and magnitude of the change in addition to the lack of change in the government SA, strongly suggest that the decrease in perinatal mortality in the icddr, b SA did occur due to the new initiative.

Notably, in the government SA, there was no decrease in perinatal mortality during the post-intervention follow-up period despite the substantial increase in positive care seeking behaviors. This change in care seeking may be explained by implementation of demand-side financing initiated in 2008 in the government SA only that offered incentives for ANC care (US$ 1.0 for each ANC visit) and facility delivery (US$ 7.7 for each delivery). In addition, a lump-sum of US$ 93.0 is offered to the public or private facility that performs a cesarean section if needed [26]. That there was no impact on perinatal mortality with this initiative warrants further exploration.

The MNCH intervention package stressed intensive preventive community-work coupled with improved quality of facility care and strengthening linkages between community and facility care. The study achieved high coverage of key process indicators such as pregnancy home visits, HBLSS sessions for pregnant women and their support persons, and immediate home-based postnatal care. The program was also successful at creating demand for key facility-based interventions through community-based activities, improving knowledge of danger signs, and recognition and referral (ANC coverage, facility delivery). That facility-based delivery increased from 48% to 72% is an encouraging achievement in a country where only 21% of women give birth in a facility nationwide [31].

The MNCH program implemented interventions known to improve perinatal health. Earlier studies had demonstrated the association of improved perinatal health with ANC [32] and birth-preparedness [33,34]. However, we didn't observe any association between ANC and perinatal mortality in the government SA, which probably reflects absence of effective linking between continuum of health system delivery modes and also lack of quality of services offered. In the icddr, b area, interventions during the intra-partum period such as prophylactic steroid use in preterm labor [35], antibiotic use for preterm rupture of membrane [36], planned cesarean section for breech, emergency obstetric care including cesarean section, and also induction of post-term pregnancy [37] have proved efficacious in improving perinatal health. Post-natal interventions such as facility-based Kangaroo Mother Care [38,39], early breastfeeding [40], and immediate postnatal visits for home births [41] have reported improvements in neonatal survival. Taken together, the decreased perinatal mortality in this study is likely attributed to the positive changes in care seeking behavior plus the availability of high quality services at community and basic emergency obstetric care levels (sub-centre and Matlab Hospital). Although improvement of quality of care was not undertaken in comprehensive emergency obstetric care facilities (these same facilities were used by both the icddr, b and government SA women), the MNCH program did improve the referral system to ensure timely response at district level (comprehensive emergency obstetric care) facilities.

There is a paucity of studies evaluating the effect of an integrated approach involving the continuum of health system delivery on fetal or neonatal health outcomes [12]. In addition to studies mentioned in the introduction section, several other studies have also evaluated packages of interventions during pregnancy and post-partum periods, resulting in 30-40% reduction of neonatal mortality. A study in Pakistan that trained traditional birth attendants ensured provision of safe delivery kits and established a link of traditional birth attendants with back-up support to improve pregnancy and intra-partum care resulted in about 30% reduction of perinatal mortality [33]. Another Pakistan study by Jokhio et al. reported about 28% reduction in neonatal mortality and 35% reduction in stillbirth with lady health visitors who provided home-based newborn care with visits during pregnancy and post-partum periods [34]. These successful community intervention packages were conducted in settings with moderate to high levels of neonatal mortality (> 30 per 1000 live births) and stillbirth rates (> 30 per 1000 births) at baseline. The only study that evaluated a package of community-based intervention in an area with low level of neonatal mortality (< 30 deaths/1000 live births) was in Mirzapur, Bangladesh; they reported no effect on perinatal mortality [15]. Considering the low rate of perinatal deaths at initiation of the integrated package (48/1000 births), the reduction in perinatal mortality observed in the present study is substantial and consistent with other studies mentioned above.

Verbal autopsy data revealed that there was substantial decrease of birth-asphyxia, infection, and other-cause related mortality. However, birth asphyxia still remains a major contributor of neonatal death. Improving the quality of care as well as care seeking has limits; other factors that impact outcomes include the time and process of decision making at the home and facility levels which also need improvement. Although we have direct contact with the district/private clinic hospitals, there was limited influence over the decision making process to provide or use specific interventions in referral facilities. For example, prompt consent for cesarean section, arrangement of blood if needed, and absence of quality immediate post-natal care in the tertiary facility level may result in poor outcomes. Delays in referral from Matlab Hospital to district level facility may also occur, especially from the low-socioeconomic strata, and result in poor outcomes in district level facilities [42]. Strikingly, there was no reduction of mortality from preterm/growth retarded deaths. This may indicate that the usual care (skin-to-skin contact) is not sufficient to improve outcomes for very preterm and/or low birth weight neonates. Further exploration is warranted to explain this finding.

The full package of intervention as shown in Figure 1 and more specifically the frequency of community interventions such as HBLSS visits, pregnancy home visits, post-natal home visits might be viewed by some as overambitious and challenging. However we believe that the infrastructure and human resources are in place in the government system that could implement a similar program. Under the current government health infrastructure and program, community health worker, Female Welfare Assistant (FWA), covers a population of about 5,000-6,000, and is to visit each household every 2 months and provides mainly family planning services. In addition, a Health Assistant (also equivalent to CHRW) is available for the same population to provide immunization from outreach clinics. Similarly, an icddr, b CHRW covers a population of about 2,700, with both family planning and immunization activities, and visits each household bimonthly. In the icddr, b SA CHRWs are also involved with ongoing studies on child and maternal health.

Since this study was carried out, the GoB has been implementing about 1,600 community clinics, each serving a 6,000 population with such services as immunization, family planning, ANC and treatment of minor illnesses, through a FWA. Prior to this new GoB program, the lowest GoB facilities were Health and Family Welfare Centers (union sub-centers covering a population of about 25,000 each) staffed by one nurse-midwife offering ANC and delivery care of pregnant women, similar to the icddr, b sub-centers. Also, the evidence-based practices during the continuum of pregnancy and post-partum periods may be readily adopted in the government sub-district level hospital as we did in the Matlab hospital. Therefore, it is not a matter that the government program lacking infrastructure or human resources but rather inadequate linkage among health care providers at different levels as well as a lack of monitoring program and effective supervision of health workers, and also inadequate knowledge about evidence-based interventions.

## Conclusion

We observed a significant reduction of perinatal mortality over a short period of time in an area of low level of mortality at baseline. The findings are robust even after adjusting for socio-demographic factors, although the possibility of residual confounding cannot be ruled out completely. We believe that the improvement in perinatal survival most likely resulted from the integration of evidence-based interventions provided over the continuum of care from pregnancy through the postpartum period. Monitoring the process and outcome indicators in "real time" including quality assurance at all level of community and basic emergency obstetric care services was also crucial. In conclusion, we showed the continuum of care approach provided through the integration of service delivery modes decreased the perinatal mortality rate within a short period of time. We recommend further testing of this model within the government health system in Bangladesh and other developing countries urgently to identify the model that can be readily replicable in the local health system in the respective country to achieve the MDG 4.

## Abbreviations

ANC: Antenatal care; CHRWs: Community health research workers; CI: Confidence interval; GoB: Government of Bangladesh; HBLSS: Home based life saving skills; HDSS: Health and demographic surveillance system; icddr, b: International Centre for Diarrhoeal Disease Research: Bangladesh; OR: Odds ratio; MCH-FP: Maternal: child health and family planning; MDG: Millennium development goal; MNCH: Maternal, neonatal and child health; SA: Service area.

## Competing interests

The authors declare that they have no competing interests.

## Authors' contributions

AR, AM, LS, MY, PKS, SEA, AB and MK contributed to the study concept and design. AR, JP, Aminur R, HB, HR, AM, and MY supervised implementation of the study. AR, MR, PKS coordinated collection of field data. AR, JP, MR, AM and DH contributed initial data cleaning and analyses. All authors participated in the data analysis and reporting stage, and have seen and approved the final draft of the report.

## Pre-publication history

The pre-publication history for this paper can be accessed here:

http://www.biomedcentral.com/1471-2458/11/914/prepub
